# Recent Advances in the Management of Acute Severe Ulcerative Colitis

**DOI:** 10.3390/jcm13237446

**Published:** 2024-12-06

**Authors:** Elaine Ong Ming San, Kassem Sharif, Konstantina Rosiou, Michael Rennie, Christian Philipp Selinger

**Affiliations:** 1 Leeds Gastroenterology Institute, Leeds Teaching Hospitals NHS Trust, St James University Hospital, Bexley Wing, Beckett Street, Leeds LS9 7TF, UK; elaine.ongmingsan1@nhs.net (E.O.M.S.); kassemsharif@gmail.com (K.S.); konstantina.rosiou@nhs.net (K.R.); michael.rennie@health.nsw.gov.au (M.R.); 2 Department of Gastroenterology, Sheba Medical Centre, Ramat Gan 5262000, Israel; 3 Faculty of Medicine, Tel Aviv University, Tel Aviv 6997801, Israel; 4 Department of Gastroenterology and Hepatology, Western Sydney Local Health District, Blacktown, NSW 2747, Australia

**Keywords:** inflammatory bowel disease, ulcerative colitis, infliximab, cyclosporine, acute severe colitis, Jak Inhibitors (JAKi)

## Abstract

Acute severe ulcerative colitis is a medical emergency requiring inpatient treatment with intravenous steroids. Approximately one-third of patients do not respond to steroids sufficiently and require medical rescue therapy. Infliximab and cyclosporine are equally effective rescue agents, though infliximab is often preferred by clinicians for ease of use and greater familiarity. The use of cyclosporine is becoming more frequent, however, in patients previously exposed to infliximab. Those patients not exhibiting an adequate response to rescue therapy require colectomy. There is increasing interest in modified medical treatment to rescue the need for surgery. Janus kinase inhibitors may provide benefits when used alongside steroids from admission or as a rescue agent, but further randomised trials are needed to clearly establish their role. Intensified dosing of infliximab when used as a rescue therapy has shown mixed results but seems sensible in patients with low albumin and high disease burden. In this review, we describe the current established treatment pathways and report newer developments and evolving concepts that may in the future improve the care of patients with acute severe ulcerative colitis.

## 1. Introduction

Ulcerative colitis (UC), one of the main types of inflammatory bowel disease (IBD), is characterized by mucosal inflammation that begins in the rectum and extends proximally in a continuous fashion [1]. The majority (40%) of patients have left-sided UC with mild to moderate disease activity. However, acute severe ulcerative colitis (ASUC) develops in at least 25% of patients during their disease course [2]. Notably, ASUC can be the initial presentation in up to one-third of UC patients, with 30–40% eventually requiring colectomy following one or more severe flares [3]. The management of ASUC has undergone significant advancements in recent years, reflecting progress in therapeutic options and clinical strategies. This review aims to provide a comprehensive evaluation of the latest developments in the management of ASUC, highlighting emerging trends and their impact on clinical outcomes.

### 1.1. Definition of ASUC

The Truelove and Witts Severity Index defines ASUC as bloody diarrhoea (≥6 stools per day) accompanied by at least one marker of systemic toxicity, including a pulse > 90 beats per minute, temperature > 37.8 °C, haemoglobin < 105 g/L, or an erythrocyte sedimentation rate (ESR) > 30 mm/h [4]. Since its original description, the use of ESR has been replaced with C-reactive protein (CRP). The CRP values vary, including >10 mg/L as defined in the IBD audit or >30 mg/L as proposed in the ECCO e-guidelines [1,5,6].

In many clinical studies, and by some in clinical practice, the Lichtiger Index score has been used as an alternative to the Truelove and Witts criteria [7]. The score includes eight criteria: the number of daily stools, nocturnal stools, visible blood in the stool as a percentage of movements, faecal incontinence, abdominal pain/cramping, general well-being, abdominal tenderness, and need for antidiarrhoeals. With scores ranging from 0 to 21 points, outcomes have been subsequently defined as <10 points on two consecutive days for clinical response and ≤3 for clinical remission [7,8].

### 1.2. Initial Assessment

**Figure 1:**
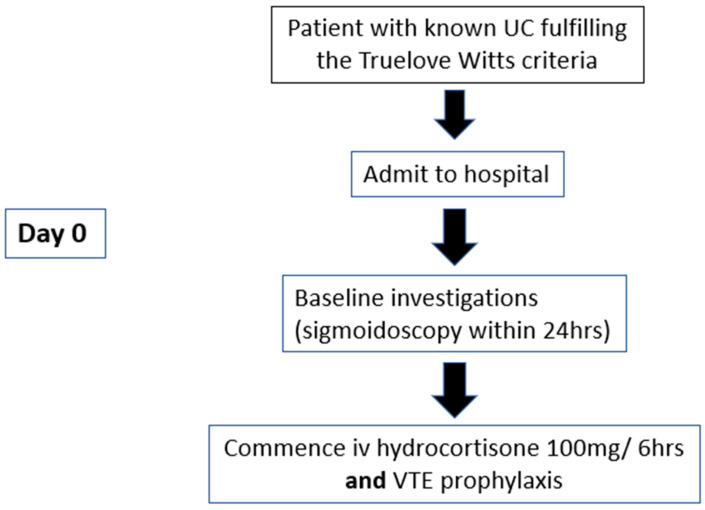
Treatment pathway for day 0.

Initial investigations for ASUC should include a full blood count (FBC), CRP, urea and electrolytes (U&E), liver function tests (LFTs), and magnesium levels. Drugs such as non-steroidal anti-inflammatories (NSAIDs) should be considered as a potential trigger. Additionally, stool samples should be collected for microscopy, culture, and sensitivity testing, as well as for Clostridium difficile antigen testing to rule out infectious causes (Figure 1) [1].

Patients with UC appear to be at greater risk for Clostridium difficile infection (CDI) due to frequent hospital visits, exposure to immunosuppressants, and altered gut microbiome [9]. The prompt recognition of CDI is crucial, as patients with concurrent UC and CDI face a heightened risk of complications. These individuals have a higher rate of requiring therapy escalation, extended hospital stays, and surgical intervention, and experience greater morbidity and mortality rates [9]. The management of CDI in UC can be challenging as symptoms may be due to both the infection and exacerbation of the underlying UC. Regardless, all UC patients testing positive for CDI should receive antimicrobial therapy.

According to the 2021 update by the Infectious Disease Society America, CDI can be classified into non-severe, severe, and fulminant [10]. Non-severe disease includes patients with a white cell count (WCC) of ≤15,000 cells/mL and a serum creatinine level < 1.5 mg/dL [10]. Severe CDI includes patients with a WCC > 15,000 cells/mL or a serum creatinine level ≥ 1.5 mg/dL [10]. Patients who present with hypotension, shock, ileus, or megacolon are classified as having fulminant CDI [10].

First-line treatment for non-severe or severe CDI includes oral vancomycin 125 mg four times daily for 10 days or fidaxomicin 200 mg twice daily for 10 days. Metronidazole is no longer recommended as a first-line therapy due to its higher failure rate [9]. For patients with fulminant CDI, treatment should include oral vancomycin 500 mg four times daily along with intravenous (IV) metronidazole 500 mg every eight hours. These patients require close monitoring to detect the development of toxic megacolon or bowel perforation, which may necessitate surgical intervention.

All patients with ASUC should have a plain abdominal X-ray (AXR) to monitor complications such as toxic megacolon and/or perforation. Toxic megacolon is defined as total or segmental colonic distension of >6 cm in the presence of systemic toxicity [11]. This represents a life-threatening complication and, in the majority of cases, requires urgent surgical intervention. Computed Tomography (CT) scans have minimal impact on the decision to perform colectomy, and thus, they are not routinely recommended [12].

An unprepared flexible sigmoidoscopy is useful to evaluate patients’ disease activity and colonic biopsies should be obtained to rule out cytomegalovirus (CMV) in all patients with ASUC. CMV is a highly prevalent virus that typically causes mild or no symptoms in immunocompetent adults. However, immunosuppressed patients, such as those with ASUC, are at significantly higher risk of reactivation, which may worsen their outcomes [13]. However, in certain cases, it is difficult to differentiate whether CMV is a trigger for exacerbation or merely an innocent bystander in severe disease [13].

While CMV positivity, identified via PCR or histopathology, is not consistently linked to more severe disease, it may still contribute to worse outcomes. Differences in outcomes, particularly between those with tissue PCR positivity and those with CMV inclusion bodies, are confounded by higher antiviral use in the latter group, making definitive conclusions challenging [13]. The role of quantitative viral load in colonic tissue is controversial due to conflicting results [13].

Some studies associate CMV positivity in ASUC with an increased need for rescue therapy, longer hospital stays, and higher readmission rates, but no significant difference in colectomy risk or colectomy-free survival [13]. However, others have reported worse colectomy outcomes and survival in patients with CMV, thus reflecting conflicting evidence on its clinical impact (8) [13]. Antiviral therapy in cases of severe steroid-resistant UC with detectable CMV is recommended with IV ganciclovir 5–7.5 mg/kg twice daily for 2 weeks [14]. In these cases, immunosuppressive therapy should not be discontinued, and steroid doses should be reduced. However, discontinuation of immunosuppression is recommended in patients who develop disseminated disease [15].

For assessment of endoscopic disease activity, the two most commonly utilised scoring systems are the Ulcerative Colitis Endoscopic Index of Severity (UCEIS) and the endoscopic Mayo score, as seen in Table 1 and Table 2. An UCEIS score of 0–1 indicates remission, 2–4 reflects mild disease, 5–6 signifies moderate disease, and 7–8 represents severe disease. Severe scores (7–8) strongly predict the need for rescue therapy or colectomy.

Additionally, a pre-immunosuppression screen for latent tuberculosis, hepatitis B, hepatitis C, human immunodeficiency virus (HIV), and varicella zoster virus should also be performed in the event that rescue therapy with cyclosporine or infliximab (IFX) is required.

## 2. Treatment

### 2.1. Corticosteroids

Since the seminal 1955 randomised controlled trial (RCT) by Truelove and Witts demonstrating the efficacy of IV corticosteroids, these medications have remained the first-line treatment for patients with ASUC [4]. Current guidelines recommend administering IV corticosteroids, equivalent to 0.8–1 mg/kg of methylprednisolone, for 5–7 days as the initial therapy in managing ASUC [17]. Prior to the introduction of intravenous steroids for the management of ASUC, patients offered very poor results with high rates of morbidity and mortality [4].

Daily assessment should include symptom evaluation such as stool frequency, urgency, and bleeding, as well as monitoring of biochemical/inflammatory markers to determine response to corticosteroid therapy. More than one-third of patients with ASUC do not respond to IV corticosteroids and approximately 20% of patients admitted with ASUC require a subtotal colectomy with a temporary end ileostomy on their first admission despite medical rescue therapy.

There are several predictive indices available to assess the likelihood of corticosteroid failure in ASUC.

The Swedish Index (or fulminant colitis index) described by Lindgren et al. calculates a 3-day score (stool frequency/day + 0.14 × CRP mg/L), with a score ≥ 8 having a 72% positive predictive value for colectomy [18].

Jain et al. reported that all patients with a UCEIS >6 and a day 3 fecal calprotectin (FCP) > 1000 µg/g failed corticosteroid therapy [19].

The Edinburgh risk score by Ho et al. evaluates mean stool frequency over the first three days of admission, colonic dilatation (>5.5 cm), and hypoalbuminemia (<30 g/L) on day 1, with a score ≥ 4 predicting corticosteroid failure with 85% sensitivity and 75% specificity [20].

Gibson et al. identified an elevated day 3 CRP-to-albumin ratio as an early predictor of steroid-refractory ASUC. Its predictive accuracy is further enhanced when combined with stool frequency [21].

**Figure 2:**
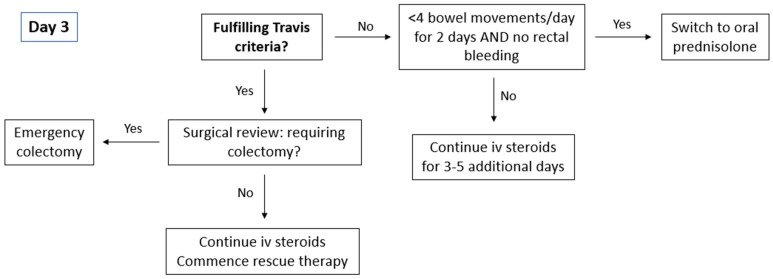
Treatment pathway for day 3.

The Travis criteria developed in 1996 (Figure 2) is the most commonly used risk stratification tool. On day 3 of corticosteroid therapy, patients with a stool frequency of >8 per day or a stool frequency of ≥3 per day combined with a CRP > 45 mg/L have an 85% likelihood of requiring colectomy during their admission. Even in the current post-biologic era, the Travis criteria have demonstrated good performance characteristics with a sensitivity of 62%, specificity of 64%, and a positive predictive value of 56% in predicting response to corticosteroid therapy [22].

**Figure 3:**
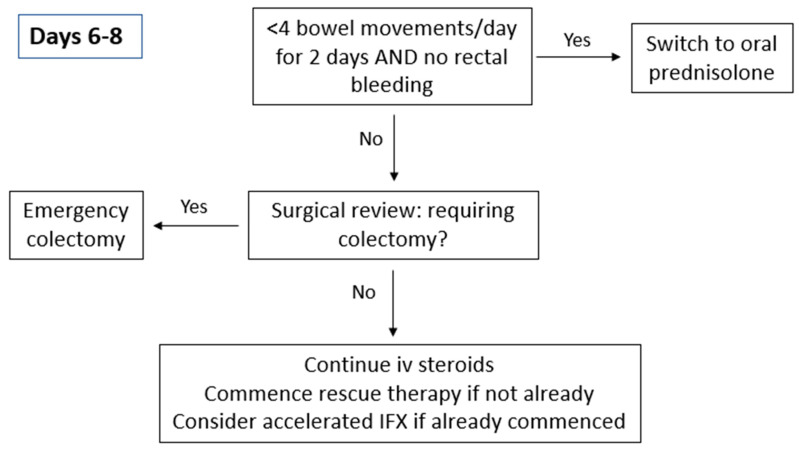
Treatment pathway day 6–8.

For patients responding to IV corticosteroid therapy (Figure 3), treatment can be extended for an additional 3 to 5 days or transitioned to oral prednisolone at 40 mg/day, with a planned tapering of 5 mg per week. A treatment strategy involving the initiation of steroid-sparing agents should be implemented during hospitalisation or shortly after discharge. Patients who do not respond adequately to corticosteroids should be evaluated for second-line medical therapies or surgical intervention. Timely decision-making is critical as delays in administering medical rescue therapy are associated with prolonged hospital admissions and delays in emergency colectomies lead to an increased risk of post-operative complications [23,24].

### 2.2. Calcineurin Inhibitors (CNIs)

Cyclosporine acts by binding to the cytoplasmic protein cyclophilin, which inhibits calcineurin, a key regulatory factor. This inhibition suppresses the production of multiple cytokines, including interleukin (IL)-2, IL-3, IL-4, tumour necrosis factor (TNF)-α, interferon-gamma, and granulocyte–macrophage colony-stimulating factor, ultimately preventing T cell proliferation and differentiation [25]. In 1994, Lichtiger et al. conducted the first trial demonstrating the efficacy of an IV dose of 4 mg/kg/day [7]. A subsequent Belgian RCT in 2003 found no additional clinical benefit of high-dose IV cyclosporine over low-dose therapy for ASUC [26]. A dose of 2 mg/kg IV cyclosporine is now recommended to reduce toxicity, with a target trough concentration of 150–250 ng/mL. Responders should transition to an oral dose at twice the IV dose, divided twice daily, with a target trough concentration of 100–200 ng/mL [27]. Oral cyclosporine should be continued for several months as bridging therapy, with better long-term outcomes achieved when thiopurine maintenance therapy is used [28,29]. However, outcomes are significantly poorer in patients requiring cyclosporine salvage therapy who have already failed thiopurine treatment [28].

In recent approaches, especially in thiopurine-experienced patients, cyclosporine is used as an induction agent, followed by biologics like vedolizumab or ustekinumab for maintenance in steroid-refractory UC. A 2019 retrospective study by Pellet et al. evaluated 39 patients with steroid-refractory UC and prior anti-TNF failure, using a combination of CNIs (mostly cyclosporine) and vedolizumab [27]. The induction of remission with CNIs and maintenance of remission with vedolizumab as a strategy avoided colectomy in approximately two-thirds of patients within a year and was generally safe [27].

Similarly, Ollech et al. conducted a larger retrospective study in the same year with 71 patients (48 treated with cyclosporine), finding 67% became colectomy-free at 12 months and 55% at 2 years [30]. This approach may benefit patients with ASUC, especially those with prior anti-TNF exposure.

Ustekinumab, though approved for severe UC, has limited evidence supporting its role as an induction therapy in ASUC [31]. In 2023, Veyrard et al. conducted a small retrospective study of 10 patients with steroid-resistant ASUC treated with calcineurin inhibitors (nine with cyclosporine) [32]. Most patients had prior exposure to IFX (90%) and vedolizumab (80%). At 6 months, no patients required colectomy, though one failed to achieve remission, and two required ustekinumab dose optimisation [32].

Despite advancements in IBD treatment, severe cases like ASUC continue to pose challenges, with many patients ultimately requiring colectomy. As prior exposure to thiopurines and anti-TNF agents is common in modern practice, alternative therapeutic strategies are essential. The evolving approach of cyclosporine as a rescue induction therapy, followed by maintenance with vedolizumab or ustekinumab, introduces a novel and promising option for managing these complex cases [31].

### 2.3. Infliximab (IFX)

Tumour necrosis factor α (TNF-α) is a key proinflammatory cytokine involved in the pathogenesis of UC. IFX, a chimeric IgG1 monoclonal antibody, binds with high affinity to TNF-α, neutralising its biologic activity.

The efficacy of IFX for UC was established in 2005 through the Active Ulcerative Trials 1 and 2 (ACT 1 and ACT 2), which demonstrated that patients with moderate-to-severe UC treated in the outpatient setting with IFX followed by maintenance every 8 weeks had significantly higher clinical response rates at weeks 8, 30, and 54 compared to the placebo [33]. In the same year, a Scandinavian RCT by Jarnerot et al. showed that a single 5 mg/kg dose of IFX as rescue therapy for ASUC significantly reduced 3-month colectomy rates compared to the placebo in patients initially treated with IV betamethasone (24) [34].

The standard induction regimen for IFX is 5 mg/kg at weeks 0, 2, and 6, and combination therapy with a thiopurine is recommended for responders, even in those who previously failed thiopurine monotherapy. Combination therapy has been shown to enhance IFX levels and reduce immunogenicity, improving treatment outcomes [35].

### 2.4. Infliximab (IFX)-Accelerated and High-Dose Therapy

Possibly due to clinician preferences, observational data on long-term colectomy rates, drug side effects, and ease of drug protocols, there have been large increases in the use of infliximab as the standard salvage therapy [36]. A recent 2021 Italian observational study of 372 patients with ASUC from 2005 to 2017 found an increase in medical salvage therapy of 14% of presenting patients over this time with the choice of infliximab increasing from 61.9% to 91.6%, with an early colectomy rate of 9.4% and a rate of colectomy of 40% at 5 years [37].

As the failure rates of infliximab in this setting remain high, attempts to optimise infliximab therapy with accelerated or higher dose induction treatment remain an area of interest. It is hypothesised that accelerated or higher dosing of infliximab may provide improved clinical outcomes compared to standard therapy. The severity of acute inflammation in ASUC presentations may lead to higher drug concentration requirements and trough levels to control for the larger TNF and cytokine burden, proteolytic degradation, low serum albumin, and monoclonal antibody loss in the diseased colon [38,39].

Retrospective observational data results for clinical outcomes of both high-dose infliximab therapy and accelerated infliximab therapy have been mixed. A 2018 systemic review of nine adult studies identified considerable heterogenicity of the observational studies, including varying definitions, disease severity, infliximab doses, and timing, as well as a lack of prospective data [40]. A single-site study from 2005 to 2013 treated 50 steroid-refractory ASUC patients with infliximab standard therapy versus accelerated therapy with three 5 mg per kg induction doses within 24 days, with a primary outcome of colectomy-free survival. Early colectomy was significantly lower (6.7% versus 40%, *p* = 0.039) in the accelerated group; however, the study was limited by sample size, provider bias, and variability in dose timing [41].

Subsequently, a retrospective study reported 146 ASUC patients with a standard 5 mg per kg (120 patients) and a high dose of 10 mg per kg (26 patients) infliximab therapy. Patients requiring an additional dose of 5 mg per kg in the first 7 days were defined as accelerated IFX dosing (AD) [42]. A total of 22 patients required AD, all from the standard therapy arm. Colectomy was highest in the AD group (41%), compared to the standard group (12.2%) and the high dose group (15.4%). This result suggests no significant difference between the high dose and standard therapy, while those requiring AD remained at high risk of colectomy despite further infliximab therapy [42].

A study at 11 UK centres involving 131 ASUC patients from 2016 to 2018 defined accelerated dosing as at least two doses of 5 mg/kg with a second dose received on or before 7 days after the first dose and/or those who received 10 mg/kg for their first dose with a further dose within 2 weeks [43]. A total of 29 patients received accelerated therapy, with propensity-matched scoring for 52 patients resulting in a 30-day colectomy rate of 57% in standard therapy vs. 27% on accelerated therapy (*p* = 0.048) without significant difference in long-term colectomy rates (57% vs. 31%, *p* = 0.09) [43]. No difference in colectomy rates (8% versus 9%) was found in a 2019 multicentre retrospective study of 213 patients with accelerated treatment versus standard infliximab treatment [44].

The only randomised controlled trial of accelerated IFX dosing was recently published in November 2024. PREDICT-UC (21) compared intensified infliximab therapy versus standard therapy as salvage treatments for steroid-refractory ASUC across 13 Australian hospitals. Between 2016 and 2021, 138 patients were enrolled and randomised, 1:2 to 10 mg per kg induction (46 patients) and standard 5 mg per kg induction (92 patients) [45]. The standard group was further randomised to accelerated treatment at 0, 7, and 21 days (48 patients) and the standard treatment at 0, 14, and 42 days (44 patients). All three treatment groups were given further salvage doses for clinical nonresponse if required. After three months, the cohort was re-randomised to maintenance therapy of thiopurine monotherapy (60 patients), combination therapy (10 patients), or infliximab monotherapy (eight patients) with 12 months follow-up. Higher dose treatment of 10 mg per kg was found to be not superior to standard dosing with no statistical difference in the primary outcome for the day 7 clinical response between groups (65% versus 61%, *p* = 0.62) [45]. Colectomy-free survival at 12 months for the high dose induction group, accelerated standard induction group, and the standard induction group was 7%, 22%, and 15%, respectively (*p* = 0.13). Successful infliximab rescue by 3 months was 83%, 81%, and 80% (*p* = 0.93). Relapse by month 3 was 26%, 15%, and 9% (*p* = 0.29). Combined clinical and endoscopic remission at 3 months was 41%, 42%, and 41% (*p* = 1.0) [45]. A post hoc analysis was completed on baseline biochemical markers on the day of infliximab induction. This identified a subgroup of patients with albumin less than 25, with a primary outcome day 7 response of 64% in the 10 mg per kg group compared to 45% in the 5 mg per kg group; however, the difference was not statistically significant (CI 0.86–2.39, *p* = 0.17). Similarly, patients with a CRP of more than 50 had a primary outcome day 7 response of 60% compared to 42% in the 5 mg per kg group (0.71–2.74, *p* = 0.34) [45].

While the biochemical and pharmacodynamic rationales for accelerated dosing of IFXC are compelling, the evidence does not support the need for routine accelerated dosing so far. However, a subgroup of ASUC patients with high CRP and low albumin may benefit from higher dose induction. This will likely be a topic of further investigation, as would targeted dose therapy, such as therapeutic drug monitoring or biomarker development. After many years of limited observational data, further prospective studies are currently ongoing, including the “TITRATE” study (NCT03937609) investigating the optimisation of infliximab in ASUC by comparing standard infliximab therapy with personalised infliximab dosing, due to be completed in 2025.

### 2.5. Cyclosporine vs. Infliximab

Two pivotal studies, CYSIF (2007–2010) and CONSTRUCT (2010–2013), evaluated the efficacy of cyclosporine and IFX in steroid-refractory ASUC [46]. Both demonstrated that the two therapies are equally effective, achieving early clinical response rates exceeding 80% within the first week and displaying acceptable safety profiles [46]. The CYSIF trial, an open-label European randomised study involving 115 patients, found no superiority of IV cyclosporine over standard induction therapy with IFX [47]. Similarly, the CONSTRUCT trial, a pragmatic study conducted in the UK with 270 patients, reported comparable quality-adjusted colectomy-free survival outcomes over 12–36 months for both agents [48].

Although IFX and cyclosporine are considered equally effective as rescue therapies for steroid-refractory ASUC, the choice should be individualised. Key considerations include comorbidities, the intended maintenance therapy post-remission, and the experience of the treating clinician and institution with each therapy [45]. IFX is the preferred option by many clinicians due to its ease of administration and familiarity with use in the outpatient setting [45]. It also allows for higher doses to be administrated, although there is currently insufficient evidence to support such practice [45].

Cyclosporine, on the other hand, is preferred in patients with tuberculosis, hepatitis B, demyelinating conditions (e.g., multiple sclerosis), or New York Heart Association Functional Classification (NYHA) 3 or 4 heart failure, as well as in thiopurine-naïve patients. Cyclosporine has a narrow therapeutic window and carries risks of adverse events, including nephrotoxicity, anaphylaxis, and infectious complications. As a result, therapeutic drug monitoring is essential, and its use as long-term therapy is limited [45].

While the primary focus in ASUC is a successful induction of remission, the anticipated choice of maintenance therapy should also guide the selection of rescue therapy. While IFX induction remains the preferred option when IFX maintenance is planned, cyclosporine induction is a more appropriate choice when transitioning to alternative biologics for long-term management [45].

### 2.6. Janus Kinase Inhibitors

Biologic drugs have transformed the management of acute severe ulcerative colitis (ASUC), providing effective options for many patients. However, challenges such as incomplete response rates, intolerance, and the development of anti-drug antibodies limit their long-term success [49]. These limitations urged the development of novel therapeutic classes that operate through alternative mechanisms. Among these emerging classes, small-molecule drugs have gained substantial attention [50]. Unlike biologics, which target extracellular cytokines or membrane-bound receptors, small molecules function intracellularly, offering a distinct mode of action.

One prominent category within this group is Janus kinase inhibitors (JAKi), which are orally administered, therefore offering advantages of convenience, rapid onset of action, as well as reduced risk of immunogenicity [51].

JAK inhibitors work by targeting the JAK and Signal Transducers and Activators of the Transcription (STAT) pathway, which plays a key role in inflammatory cytokine signalling. The JAK family consists of four distinct kinases, JAK1, JAK2, JAK3, and TYK2, and JAKi vary in their selectivity for blocking these receptors [52]. Tofacitinib, a pan-JAK inhibitor, mainly inhibits JAK1 and JAK3, with less activity against JAK2 and TYK2 [53]. It was the first JAK inhibitor approved for treating UC. Upadacitinib, on the other hand, selectively inhibits JAK1, modulating several cytokine pathways involving proteins like IL-2, IL-4, IL-7, IL-9, IL-15, IL-21, and type I and II interferons [54]. This drug is approved for both UC and CD [55]. Similarly, filgotinib, another JAK1-selective inhibitor, has also been approved for UC [55].

The efficacy of tofacitinib and upadacitinib in both the induction and maintenance of remission in ulcerative colitis has been well demonstrated in numerous randomised controlled trials, the details of which are beyond the scope of this review [56,57]. In a post hoc analysis, the rapidity of symptomatic improvement was assessed in patients with moderately to severely active ulcerative colitis who had previously exhibited inadequate response, loss of response, or intolerance to corticosteroids, Azathioprine, 6-mercaptopurine, and/or anti-TNF therapies (infliximab and adalimumab) within the OCTAVE Induction 1 and 2 trials [56]. A significant reduction in the Mayo stool frequency subscore was observed with tofacitinib 10 mg twice daily compared to the placebo within just 3 days. Similarly, improvement in the Mayo rectal bleeding subscore with tofacitinib reached statistical significance by day 3, with continued improvement each day through day 15. A comparable trend was noted in the reduction of daily bowel movements. For context, in ASUC, infliximab showed an improvement in the Lichtiger index within 3 days compared to cyclosporine; however, this effect was not sustained beyond day 4 [47]. In vedolizumab induction trials, significant improvement was demonstrated only by weeks 4–6 [58,59].

In a similar post hoc analysis conducted in the U-ACHIEVE and U-ACCOMPLISH trials [57], patients treated with upadacitinib showed significant improvements compared to the placebo in all UC symptoms starting between days 1 and 3, with effects sustained through day 14. Additionally, there were increased rates of clinical remission and response based on the partial Mayo score from week 2, along with significant quality of life improvements at weeks 2 and 8 [60].

Especially in the context of ASUC, early promising data have been published demonstrating the efficacy of tofacitinib. However, these findings are primarily limited by the studies’ nature, being uncontrolled case series with small sample sizes [61,62,63]. Moreover, variability among the studies was noted, with some using 10 mg three times daily [64], 10 mg twice daily [62], 15 mg twice daily [61], and others combining tofacitinib with cyclosporine [65].

An uncontrolled observational study by the GETAID-TALC group reinforced the clinical efficacy of tofacitinib as rescue therapy in patients hospitalised with UC flares [66]. With a median follow-up of 6.5 months, the study included 55 patients, 49 of whom had experienced prior infliximab failure, and 19 had previously been treated with ciclosporin. In this cohort, 65% of patients were on concomitant steroids at the initiation of tofacitinib, and 14.5% were treated with ciclosporin during the current flare. Colectomy-free survival was approximately 75% at both 3 and 6 months. Rates of clinical response, clinical remission, and steroid-free clinical remission at week 6 were 60%, 45.5%, and 37.5%, respectively, underscoring the potential of tofacitinib as a promising therapeutic option [66]. In terms of safety, no deaths were observed, and two cases of herpes zoster infections were reported in patients over 60 years of age but no venous thrombotic events or major adverse cardiovascular events were recorded [66].

In a retrospective case-control study involving biologic-experienced patients hospitalised with ASUC and initiated on tofacitinib, the tofacitinib group had a higher proportion of previous biologic use and a longer duration of prior corticosteroid use compared to sex- and date-matched controls. Both groups received concomitant intravenous corticosteroids as part of the standard care for ASUC. Notably, two patients in the control group received cyclosporine rescue, while 40% received infliximab rescue. None of the patients in the tofacitinib group received rescue therapy with infliximab or cyclosporine. Among the tofacitinib group, 40% received tofacitinib 10 mg BID, and 60% received 10 mg TID. Tofacitinib induction therapy, when combined with intravenous corticosteroids, was associated with a reduced risk of colectomy at 90 days compared to controls (hazard ratio = 0.28, 95% CI 0.1–0.81, *p* = 0.018). In subgroup analysis, this benefit was statistically significant only at the 10 mg TID dose and not at the 10 mg BID dose, although it was limited by the small sample size, which may have affected statistical power [67].

A recent single-centre, double-blind, placebo-controlled trial compared tofacitinib 10 mg TID to the placebo over a 7-day period, with both groups continuing intravenous corticosteroids. Of the 104 participants recruited, 54 were randomly assigned to receive tofacitinib. By day 7, treatment response was observed in 83% of patients in the tofacitinib group, compared to 59% in the placebo group (odds ratio 3.42, 95% confidence interval 1.37–8.48, *p* = 0.007). The tofacitinib group also had a significantly lower need for rescue therapy by day 7. There were, however, serious adverse events in both groups, including four deaths and one case of dural deep sinus thrombosis [68].

Upadacitinib, more recently approved for the treatment of UC, likely exhibits similar efficacy tendencies, though the current literature is limited by the same constraints as the early uncontrolled case series for tofacitinib. In a case series of 25 patients with ASUC treated with upadacitinib, only 25% required colectomy. Among the 18 patients with available data who did not undergo colectomy, 83% achieved steroid-free clinical remission.

Taken together, the growing body of evidence suggests that JAK inhibitors hold significant promise as therapeutic options in the management of ASUC, particularly for their rapid onset of action and potential to achieve clinical remission. It remains unclear whether JAKi should be positioned as an adjunct to intravenous steroids from admission or whether a placement as rescue therapy after failure to respond to 3 days of steroids is preferable. Defining optimal dosing, assessing long-term safety, and clarifying whether JAKi should be used as monotherapy or in combination with other agents are key areas for future investigation. As more data emerge, JAKi could potentially establish a vital role in the acute care of ASUC, offering an effective and convenient alternative for patients in need of rapid and sustained relief. The potential role of rescue therapy should be explored in a head-to-head trial against cyclosporine or infliximab.

### 2.7. Recent Advances in ASUC: IASO Trial

The cytokine family of IL-1 has recently been investigated as a possible novel treatment target in ASUC. IL-1β activity is a significant mediator in autoinflammatory responses via its role in disturbing the intestinal barrier epithelium and mediating the differentiation and activation of Th17 cells [69]. During ulcerative colitis flares, the activation of Toll-like receptors and NOD-like receptors leads to the formation of the NLRP3 inflammasome within the colonocyte cytosol, activating the enzyme caspase-1 [69]. Pro IL-1β is then cleaved into its active state of IL-1β by caspase-1, leading to an extracellular inflammatory cascade within the lamina propria along with TFN-a, IL-6, IL-18, and subsequent T cell recruitment and activation. IL-1β released by immune cells is also promoted by dysbiosis in the gut, further stimulating a pro-inflammatory state [69].

Anakinra (which inhibits IL-1β) has been effectively used in other autoinflammatory conditions that are driven by the inflammasome and IL-1, such as gout, and the use of Anakinra for the control of active ulcerative colitis was investigated in murine models, showing histological distal colon improvement and decreased pro-inflammatory levels of CD8 T cells [70]. The interleukin 1 blockade in an Acute Severe Colitis (IASO) phase II trial was a double-blinded randomised 1:1 controlled trial with Anakinra versus a placebo for ASUC in addition to standard steroid therapy [71]. The primary outcome of the need for rescue medical therapy with infliximab or cyclosporin or surgical salvage within 10 days of commencement of IV corticosteroids was not met and there was no statistical significance between the two groups despite rescue therapy being required in 43% of the Anakinra group compared to 26% in the placebo group [72]. The need for colectomy by day 98 was 11% in the Anakinra group compared to 4% in the placebo group. The study was subsequently discontinued for futility [72]. This was the first prospective trial for Anakinra therapy in ASUC, and while the discontinued trial may suggest that IL-1 is not a therapeutic target, further novel studies are required to identify potential new treatment targets.

### 2.8. Sequential Treatment in ASUC

The current guidelines advocate against sequential therapy (cyclosporine in patients who have failed infliximab and vice versa) due to concerns regarding complications linked to immunosuppression and worse surgical outcomes due to delayed surgery in sick patients [1]. Only a few studies have evaluated the efficacy and safety of sequential therapy. In a small study of 19 patients, 40% of patients receiving infliximab after cyclosporine failure and 33% of patients receiving cyclosporine after infliximab failure achieved remission [73]. However, adverse events occurred in 16% of patients, including herpetic oesophagitis, pancreatitis, and bacteraemia, as well as one death due to sepsis [73].

Another multicentre study by Chaparro et al. studied the efficacy and safety of infliximab after cyclosporine failure. The study included 47 patients, of which 35 completed infliximab induction. Among the 35 patients who received three infliximab infusions, 60% went into remission and 37% had a partial response [74]. The colectomy rate in this study was 30%; therefore, the authors concluded that the effectiveness of infliximab in avoiding colectomy is relatively high. However, the incidence of adverse events was still the main concern regarding sequential treatment, as they occurred in 23% and included one death from pneumonia after colectomy [74].

Moreover, in a multicentre study in France by Leblanc et al. including 86 patients who received sequential therapy for ASUC, the probability of colectomy-free survival was 61.3 ± 5.3% at 3 months and 41.3 ± 5.6% at 12 months. Adverse events included nine infectious complications and one death due to pulmonary embolism on day 1 post-surgery [75].

Narula et al. conducted a systematic review of 314 patients who received sequential therapy [76]. This approach was found to be associated with a clinical response rate of 62.4%, clinical remission rate was 38.9%, and colectomy rates at 3 months and 12 months were 28.3% and 42.3%, respectively [76]. A risk of serious infections of 6.7% and 1% risk of mortality was found, somewhat lower than reported in initial studies [76]. However, the results need to be carefully taken into consideration due to the low quality of data included in this systematic review.

Taken together, sequential therapy with infliximab following the failure of a calcineurin inhibitor or vice versa should best be avoided not only due to the risk of adverse events but also because this strategy might further delay needed surgery in acutely unwell patients. If sequential treatment is chosen, then this should be reserved for specialised centres under close monitoring and after careful counselling of the patients about the risks.

## 3. Maintenance Treatment Following Successful Induction of Remission with Steroids in ASUC

**Figure 4:**
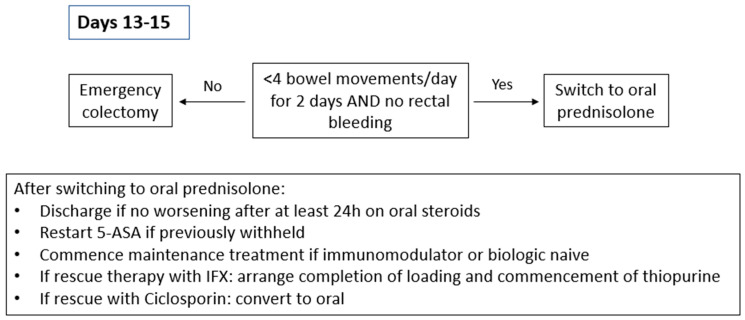
Treatment pathway days 13–15.

At present, there are limited data to guide the choice of maintenance treatment in patients with ASUC that have responded to steroids. In biologic-naïve patients, commencing 5-aminosalicylates and/or a thiopurine could be considered. In fact, the current BSG guidelines suggest commencing Azathioprine in patients who have responded to intravenous steroids (Figure 4) [1]. Equally, first, presentation with ASUC or admission with ASUC could be considered indicative of an aggressive phenotype of the disease, and early treatment with advanced therapies could be advocated [35].

In a retrospective study in Italy of 204 patients admitted with ASUC who responded to intravenous steroids, 106 patients were prescribed aminosalicylates, 72 patients received immunomodulators, and the remaining 26 patients received combination therapy with infliximab and thiopurine. After propensity score matching, no difference was observed between the three groups, suggesting that 5-ASA could be prescribed in treatment-naïve patients responding to steroids [37]. However, it is worth noting that up to 50% required escalation in the long term.

Another retrospective study by Salameh et al. included 142 patients after induction of remission with steroids, of which 59 were treated with 5-aminosalicylic acid, 60 with immunomodulators, 18 with anti-TNF, and five with vedolizumab. Multivariate analysis showed that patients with <6 liquid stools per day on day 3, a partial Mayo score < 2 on day 5, and maintenance therapy with anti-TNF (HR 0.37, 95% CI [0.16–0.87]) were less likely to relapse during follow-up, suggesting that early response to steroids but also maintenance treatment with biologics are associated with more favourable outcomes in the longer term [77].

Data from a further multicentre study in France support the early use of biologics after induction with steroids in ASUC patients. Amiot et al. randomised 64 patients to receive infliximab plus Azathioprine versus Azathioprine. Combination therapy was more effective than AZA alone in preventing treatment failure, and hence, the authors conclude that this strategy should be encouraged in ASUC patients responding to steroids. Moreover, no significant differences in adverse events were observed amongst the two groups [78].

Further trials are required the determine the optimal maintenance treatment for patients with ASUC that have responded to intravenous steroids, and currently, treatment choice should be evaluated individually for each patient. However, there is growing evidence that these patients might benefit from early initiation of advanced treatments.

## 4. General Medical Management

Patients with ASUC should be admitted to the hospital under the care of a gastroenterology team, working collaboratively with a multidisciplinary team that includes colorectal surgeons, gastroenterology nurses, dietitians, pharmacists, and stoma therapists [1,35]. In addition to drug therapy, key aspects of care include thromboprophylaxis, nutritional support, thorough assessment of fluid status, and correction of electrolyte imbalances.

IBD is a well-established independent risk factor for both incident and recurrent venous thromboembolism (VTE). Patients with active IBD are estimated to have a 2–3 times higher risk of VTE compared to those without IBD [35]. Clinical factors that further increase VTE risk in IBD patients include active and extensive disease, colorectal surgery, hospitalisation, pregnancy, and corticosteroid use. As such, thromboprophylaxis should be routinely prescribed for all at-risk patients to prevent VTE [35].

The nutritional status of patients with ASUC should be assessed, ideally by a trained dietitian, and nutritional support should be provided for malnourished individuals [1,35]. Routine parenteral nutrition has no proven benefit and is associated with more complications compared to enteral nutrition, which is considered safer and more appropriate. Additionally, total bowel rest does not improve outcomes in ASUC patients and is therefore not recommended [35].

Exclusive enteral nutrition (EEN) is a nutritionally complete liquid diet provided orally or via a nasogastric tube, delivering all of a patient’s nutritional requirements. EEN may be considered as an adjunct in the management of ASUC. A 2021 RCT involving 62 ASUC patients demonstrated that EEN for 7 days, when combined with IV corticosteroids, significantly reduced corticosteroid failure rates in the per-protocol analysis (19% vs. 43%, *p* = 0.04) [79]. The EEN group also showed notable benefits in secondary outcomes, including shorter hospital stays, improved day 7 albumin levels, and reduced CRP and FCP levels [79]. A 2024 study by Bajaj et al. further highlighted the benefits of EEN-conjugated corticosteroid therapy, showing that it was associated with beneficial gut microbial changes, which correlated with enhanced clinical responses in ASUC patients [80]. Further larger studies are needed to confirm the role of EEN in ASUC.

The ECCO guidelines recommend iron supplementation for all IBD patients diagnosed with iron deficiency anaemia (IDA), with IV iron as the preferred first-line treatment in those with active disease [81]. IV iron is more effective and produces a faster response compared to oral supplementation, particularly since active inflammation impairs iron absorption [81].

Patients with ASUC require careful monitoring of their fluid status, with IV fluids provided to manage hypovolemia as required. Electrolyte imbalances, such as hypokalaemia and hypomagnesemia, should be addressed to prevent colonic dilatation.

## 5. Surgery

Emergency surgery in ASUC is indicated for cases of medical refractory disease, uncontrolled sepsis, colonic perforation, or toxic megacolon [82]. Prompt surgical consultation and early discussions about the timing and risks versus benefits of surgery are essential to minimise postoperative complications from delayed referrals. Current guidelines recommend surgery for ASUC patients who are not suitable for rescue therapy or have not responded within 7 days of rescue therapy [1,35]. However, evolving treatment options and varying patient response times have made decision-making increasingly complex, requiring close, day-by-day assessments (24) [35]. Effective decision-making involves timely risk evaluation, continuous monitoring of treatment response, and multidisciplinary team management.

In an acute setting, a subtotal colectomy with ileostomy is conducted to avoid pelvic dissection, thereby reducing morbidity and preserving options for future restorative procedures [28]. The rectal stump is either brought out as a mucous fistula or closed within the subcutaneous fat to minimise the risk of rectal stump dehiscence [82]. However, the efficacy of these techniques in laparoscopic procedures remains uncertain, and surgical decisions should be based on intraoperative findings, rectal health, and the surgeon’s expertise [82].

In those wishing to restore intestinal continuity with an ileal pouch–anal anastomosis (IPAA) in the second stage, performed preferably after three to six months, an ileal pouch is created with a de-functioning loop ileostomy, which is subsequently closed in the final stage [28]. Although IPAA provides the potential for restored bowel function, it can significantly impact the quality of life, with frequent complications such as leakage, sexual dysfunction, soiling, and pouchitis, which occurs in up to 50% of patients [28]. Female fertility can also be deeply affected, particularly in women of childbearing age. However, laparoscopic techniques may mitigate these effects by reducing intra-abdominal adhesions [28].

## 6. Conclusions

After a couple of decades where the pathway of ASUC has not changed much, there is now a growing body of evidence that will translate to changes in the pathway in years to come. In the meantime, thorough assessment and monitoring of patients with ASCU is key. Intravenous steroids and critical assessment of response after 3 days are paramount. Rescue therapy with infliximab and cyclosporine is equally effective, but around 10–20% of patients require a colectomy. The role of accelerated infliximab and JAK inhibitors will become clearer with ongoing studies due to reports in the next 2–3 years. Until then, clinicians need to focus on strict implementation of the current pathway and avoid delays in treatment, which in turn worsen outcomes.

## Figures and Tables

**Table 1:** Ulcerative Colitis Endoscopic Index of Severity (UCEIS) [16].

Vascular Pattern	Normal vascular pattern with arborisations of capillaries clearly defined	0 = Normal
	Patchy obliteration of vascular pattern	1 = Patchy obliteration
	Complete loss of vascular pattern	2 = Complete loss of vascular pattern
Bleeding	No visible blood	0 = None
	Spots or streaks of coagulated blood in the mucosa surface that can be washed off	1 = Mucosal
	Some free liquid blood in lumen	2 = Luminal mild
	Frank blood in the lumen or visible oozing from the mucosa after washing or visible oozing from a haemorrhagic mucosa	3 = Luminal moderate or severe
Erosions and Ulcers	Normal mucosa, no visible ulcers or erosions	0 = None
	Small defects in the mucosa ≤ 5 mm, white or yellow, flat edge	1 = Erosions
	Large defects in the mucosa > 5 mm, discrete fibrin covered, remain superficial	2 = Superficial ulcers
	Deeper excavated defects in the mucosa with a slightly raised edge	3 = Deep ulcers

**Table 2:** MAYO endoscopic score.

MAYO 1	Erythema, decreased vascular pattern, mild friability
MAYO 2	Marked erythema, absent vascular pattern, friability, erosions
MAYO 3	Spontaneous bleeding, ulcerations

## Data Availability

Not applicable.
